# Circulating serum HBsAg level is a biomarker for HBV-specific T and B cell responses in chronic hepatitis B patients

**DOI:** 10.1038/s41598-020-58870-2

**Published:** 2020-02-04

**Authors:** Jin Hyang Kim, Alip Ghosh, Natarajan Ayithan, Sara Romani, Arshi Khanam, Jang-June Park, Rene Rijnbrand, Lydia Tang, Michael J. Sofia, Shyam Kottilil, Chris B. Moore, Bhawna Poonia

**Affiliations:** 1 Arbutus Biopharma Corporation, 701 Veterans Circle, Warminster, Pennsylvania 18974 United States; 2 Institute of Human Virology, University of Maryland School of Medicine, Baltimore, MD 21201 United States

**Keywords:** Immunology, Viral infection

## Abstract

Chronic hepatitis B (CHB) infection functional cure is defined as sustained loss of HBsAg and several therapeutic strategies are in clinical development designed to pharmacologically reduce serum HBsAg, break immune tolerance, and increase functional cure rates. However, little is known about pre-treatment HBsAg levels as an indicator of HBV immune potential. Here, we compared the phenotypes and HBV-specific response of lymphocytes in CHB patients stratified by serum HBsAg levels <500 (HBs^lo^) or >50,000 IU/ml (HBs^hi^) using immunological assays (flow cytometry, ICS, ELISPOT). HBs^hi^ patients had significantly higher expression of inhibitory PD-1 on CD4^+^ T cells, particularly among TEMRA subset, and higher FcRL5 expression on B cells. Upon HBcAg(core) or HBsAg(env)-stimulation, 85% and 60% of HBs^lo^ patients had IFNγ^+^TNFα^+^ and IFNγ^+^ IL2^+^ CD4^+^ T cell responses respectively, in comparison to 33% and 13% of HBs^hi^ patients. Checkpoint blockade with αPD-1 improved HBV-specific CD4^+^ T cell function only in HBs^lo^ patients. HBsAg-specific antibody-secreting cells (ASCs) response was not different between these groups, yet αPD-1 treatment resulted in significantly higher fold change in ASCs among patients with HBsAg <100 IU/ml compared to patients with HBsAg >5,000 IU/ml. Thus, serum HBsAg correlates with inhibitory receptor expression, HBV-specific CD4^+^ T cell responses, and augmentation by checkpoint blockade.

## Introduction

Chronic hepatitis B infection (CHB) is a significant global health burden, represented by over 257 million infections worldwide and a high mortality due to liver cirrhosis (LC) and hepatocellular carcinoma (HCC). The current standard of care, including nucleot(s)ide analogue (NA) or peg-IFN fails to achieve the recommended curative endpoint (sustained HBsAg seroclearance) in the majority of CHB patients despite long duration of treatment^1,2^. This is because the current first-line therapy does little to remove circulating HBsAg driven by transcriptional activity of covalently closed circular DNA in infected hepatocytes, despite DNA suppression (up to 93%), ALT normalization in HBeAg-negative patients (up to 76%), and HBeAg loss in HBeAg-positive patients (up to 36%)^1,3^. Along with persistence of viral antigens, impaired HBV-specific immunity contributes to chronicity of infection^4,5^. These include a depletion or functional impairment of HBV-specific T cells with sustained expression of inhibitory receptors (PD-1, CTLA-4, TIM-3), resulting in defective proliferation and cytokine production^6,7^, and an impaired production of antibodies against HBsAg, with an expansion of atypical memory B cells^8,9^. Findings from lymphoma patients experiencing HBV re-activation due to chemotherapy (up to 26%) further reinforces the non-redundant protective role of HBV-specific lymphocytes^10,11^. While the importance of host immunity for HBV clearance is well recognized, the critical immunological events required to achieve functional cure remain largely unknown.

Persistent high HBsAg levels along with dysfunctional immunity of CHB patients has raised the possibility that HBsAg itself is immunoregulatory in HBV infection^12^. Alternatively, chronic exposure to high levels of HBsAg may render HBV-specific immune cells overly activated and functionally tolerized^13,14^. Thus, decreasing serum HBsAg by pharmacological agents could be a valuable therapeutic strategy, due to its potential to relieve immune cells from functional exhaustion and confer immune control. Accordingly, several therapeutic agents including small interfering RNA (siRNA) and nucleic acid polymers (NAPs) are in clinical development but to date there has been no direct evidence to date to support this hypothesis. Studies addressing the relationship between serum HBsAg levels and HBV-specific immune responses would provide further insights on the immunobiology of HBV and help interpret upcoming clinical trial data. Further, it is unknown whether HBsAg removal alone will be enough to induce HBV immune control or whether additional immune activation are required to achieve functional cure. In recent years, quantification of serum HBsAg has been shown to be useful for predicting disease activity, viral control status and monitoring treatment responses^15^. The utility of baseline serum HBsAg levels as a predictor for the cumulative risk for LC or HCC progression in treatment-naïve patients has also been documented^16^. In particular, very low HBsAg levels (<100 IU/ml) were shown to be associated with spontaneous HBsAg clearance^17^. More practical use of HBsAg quantification has been shown during peg-IFN therapy such that on-treatment serum HBsAg levels provide guidance for treatment outcomes^18,19^. While definitive evidences as to whether serum HBsAg levels can be a direct correlate of the immune functions of CHB patients have been lacking, a few studies have addressed the relationship between viral components and specific immune parameters. These include viral HBV DNA levels inversely correlating with the frequency of HBV-specific CD8^+^ T cells in both peripheral blood and liver^4,6,20^ and recent studies focusing on the humoral immunity of CHB patients (HBsAg-specific B cell frequency) not directly correlating with serum HBsAg levels^8,9^. Therefore, comprehensive understanding as well as the extent of the dysregulation of HBV-specific immune dysfunction associated with HBsAg levels is still warranted. To this end, we assessed the phenotype of immune cells and the responses of both HBV-specific T and B cells in CHB patients stratified by serum HBsAg levels. Finally, we evaluated if the functional response of lymphocytes to immune checkpoint blockade could be differentiated by serum HBsAg levels.

## Materials and Methods

### Patient cohort

CHB patients (n = 39) from Institute of Human Virology, School of medicine, University of Maryland, Baltimore, Maryland, USA were included in the study. Healthy donors (HD; n = 10) were recruited for comparison. Demographic details for all the study subjects are detailed in Supplementary Table 1. All patients displayed clinical, biochemical, and virologic evidence of chronic HBV infection (plasma HBsAg levels positive for more than 6 months with anti-HBcAg antibody (anti-HBc) positive), with fluctuating levels of ALT (ranging from 9 to 542 U/liters) and HBV DNA (ranging from 1.3 to 8.23 log copies/ml). Patients were from diverse ethnicities and majority of them were HBeAg (−). HBV genotypes were mostly unknown. Patients were divided based on their serum HBsAg level; the HBs^hi^ group for HBsAg >50,000 IU/ml (n = 16) and the HBs^lo^ group for HBsAg <500 IU/ml (n = 23). For some studies, patients from another cohort were divided into HBsAg >5,000 IU/ml (n = 23) and HBsAg <100 IU/ml (n = 8) based on sample availability (Supplementary Table 1). The study protocol was approved by the Institutional Review Board at the University of Maryland, Baltimore and all subjects gave written, informed consent. Peripheral blood was obtained, and PBMCs were isolated using Ficoll-Paque (Sigma-Aldrich) density gradient centrifugation. Cells were subsequently resuspended in 90% fetal bovine serum (FBS, Gibco) plus 10% DMSO and stored in the vapor phase of liquid nitrogen.

### HBsAg quantification

Serum HBsAg levels were quantified by HBsAg CLIA kit (AutoBio Diagnostic Co. Ltd) following manufacturer’s instructions. HBsAg quantification was determined from a standard curve plotted with positive controls of known concentrations from 0 to 250 IU/ml. The kit has a sensitivity less than 0.15 ng/ml. Diluted samples (1:1, 1:10, 1:100, 1:1000, and 1:10000 with PBS) from 100 μl serum were used for HBsAg quantification. Chemiluminescence was detected in Synergy H1 plate reader (BioTek Instruments, Inc.).

### Antibody staining and Flow cytometry

Phenotypes of PBMCs were analyzed using antibody staining for 30 min at 4 °C and flow cytometry acquisition (BD LSRII; BD Biosciences) with combinations of the following anti-human monoclonal antibodies: αCD3-AF700 (UCHT1), αCD3-FITC (OKT3), αCD4-PE/Cy7 (OKT4), αCD4-BV421 (OKT4), αCD8-BV510 (SK1), αCCR7-FITC (G043H7), αCD19-BV510 (HIB19), αCD21- PE/Cy7 (Bu32), αCD27-APC (M-T217), αPD-1-BV421 (EH12.2H7), αCD244(2B4)-PerCP/Cy5.5 (C1.7), αPD-L1-PE (MIH2) all purchased from BioLegend and αCD45RO-APC-eFluor780 (UCHL1) from eBiosciences. Cytokine production from T cells upon stimulation with overlapping peptides of HBV was used to assess their functional capacity. Production of IFNγ, TNFα and IL-2 was measured by intracellular staining with αIFNγ-APC (4S.B3), αTNFα-PE (MAb11) and αIL-2-PerCP/Cy5.5 (MQ1-17H12) from BioLegend. Live/dead cell discrimination was performed using Zombie NIR fixable viability Kit (BioLegend). Cells were permeabilized with Cytofix/Cytoperm (BD Biosciences) and fixed in 1% paraformaldehyde. Data were acquired and compensated using FACS Diva software (BD Biosciences) and analyzed with FlowJo (version 10).

### HBV-specific T cell response

To measure antigen-specific cytokine production, PBMCs were cultured at 37 °C in a 5% CO_2_ incubator for 10 days in complete RPMI-1640 medium at a final concentration of 1 × 10^6^/ml in the absence or presence of overlapping peptides (1 μg/ml) of HBcAg (PM-HBV-CPULTRA) or HBsAg (PM-HBV-LEPULTRA) from JPT Peptide Technologies GmbH. The genotypes of HBV from our patients were mostly unknown. To accommodate diverse HBV genotypes of patients, we used the ULTRA peptide libraries from JPT, which encompass broad spectrum of HBV sequence diversity. PM-HBV-CPULTRA contains 155 overlaping peptides (15meric) with average coverage of 92.5% of all HBV genotypes and PM-HBV-LEPULTRA contains 216 overlapping peptides (15meric) with average coverage of 83% of all HBV genotypes. Recombinant IL-2 (20 IU/ml, Tecin, Biological Resources Branch, NIH) was added at days 1, 4 and 7. For checkpoint blockade study, αPD-L1 (1 µg/ml, clone MIH1, eBioscience) were added along with HBV peptide. PBMC were re-stimulated with overlapping peptides on day 9 and Brefeldin A and Monensin (BD Biosciences) were added after 1 hr of re-stimulation and the cell cultures were continued for an additional 16 hrs. Cells incubated without HBV peptides served as a negative control. IL-2 was still added at days 1,4 and 7 to support cell survival of negative control. Cells were then stained with surface markers and intracellular cytokines for flow cytometry and data was acquired and analyzed as described above. For ELISpot analysis of total T cells secreting IFN*γ*, PBMCs were cultured with overlapping peptides (1 μg/ml) of HBcAg and HBsAg with or without αPD-L1 on pre-coated ELISpot plate for overnight. Spots representing IFN*γ*-secreting cells were developed following manufacturer’s instruction (Mabtech) and analyzed using ImmunoSpot image analyzer (Cellular Technology Ltd.).

### Soluble PD-1 and PD-L1

Plasma samples collected during PBMC separation were used to measure sPD-1 and sPD-L1, using Milliplex Map Human Immuno-Oncology Checkpoint Protein Panel (Millipore Sigma) and FLEXMAP 3D (Luminex Co.) according to manufacturer’s instructions.

### HBV-specific B cell response

B cell ELISpot assays were performed according to standard protocol from previous reports^21^. In brief, frozen PBMCs were thawed and rested overnight at 37 °C in 5% CO_2_. Cells were cultured with TLR7/8 agonist R848 (1 µg/ml, Mabtech) and recombinant-human IL-2 (rIL-2, 10 ng/ml, Mabtech) at 37 °C, 5% CO_2_ for 5 days. For checkpoint blockade study, αPD-1 (1 µg/ml, Opdivo) were added along with R848 and human rIL-2. Cells were washed twice with complete RPMI-1640 and added into ELISpot plate at 25,000 cells for total IgG and negative controls and 100,000–500,000 cells for HBsAg-specific IgG response, followed by 3-fold dilutions. For ELISpot assay, sterile 96-well multiscreen-IP filter ELISpot plate with PVDF membrane (Millipore Sigma) was coated with either αhuman capture IgG (5 μg/ml, Mabtech), recombinant HBsAg (10 μg/ml, Fitzgerald, HBV subtype adw) or PBS alone (as negative controls) for overnight at 4 °C and then blocked with 5% BSA. After incubation with cells for 18 h at 37 °C, 5% CO_2_, the plates were washed twice with 1x PBS/Tween 20, incubated with Biotin-SP-conjugated αhuman IgG (1:1000, Jackson ImmunoResearch) for 1 h, and then AP-conjugated streptavidin (1:3000, Southern Biotech) for 1 h at room temperature. Spots representing ASCs were developed using Vector blue-AP substrate kit (Vector lab) and analyzed with ImmunoSpot image analyzer (Cellular Technology Ltd.). Numbers of HBsAg-specific IgG and total IgG secreting ASCs/well was calculated by subtracting background spots from negative control wells. Data is represented as number of ASCs/10^6^ PBMCs.

### Data analysis

Statistical testing was performed using GraphPad Prism v5.0 software. For hypothesis testing among the HBs^hi^, HBs^lo^ and HD groups, One-way-ANOVA or Kruskal-Wallis with Bonferroni or Dunn’s post-hoc tests for multiple comparisons were performed for parametric or non-parametric data, respectively. Pairwise comparisons were analyzed using the paired t test or Wilcoxon signed-rank test for parametric or non-parametric data, respectively. The differences between unpaired groups were compared with unpaired t-test or Mann–Whitney U test for parametric or non-parametric data respectively. Association between serum HBsAg levels and responses to HBV specific peptide stimulation were verified by χ^2^-test on 2 × 2 contingency table.

## Results

### Frequency of immune cell subsets is independent of serum HBsAg levels

**Figure 1:**
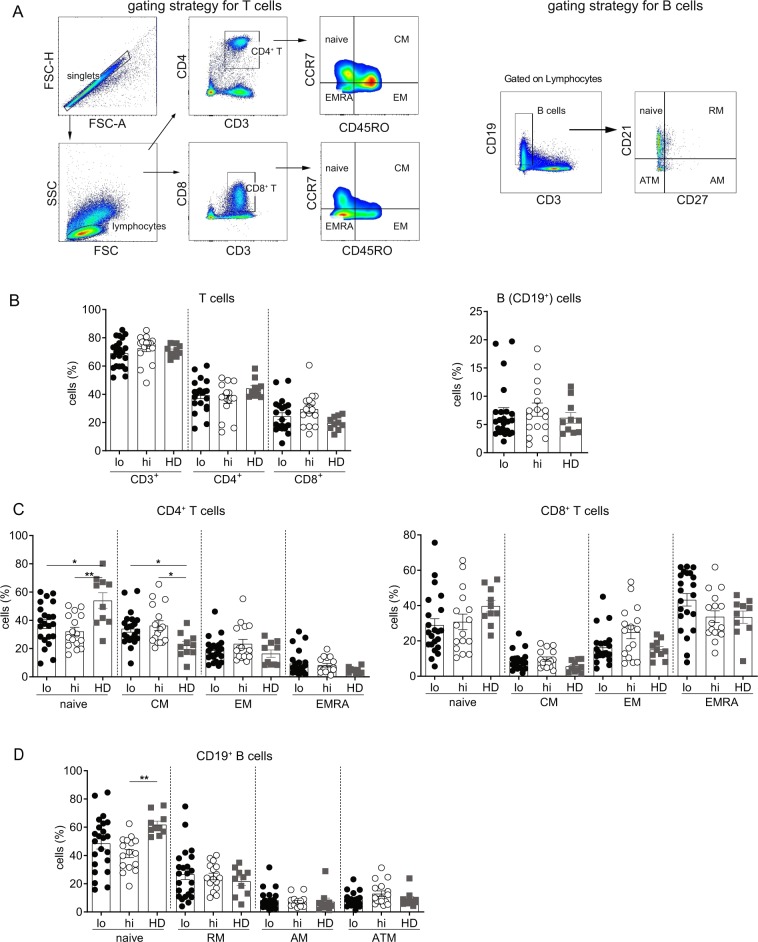
Frequencies of different immune cell subsets among the HBs^lo^ (<500 IU/ml, designated as lo), HBs^hi^ (>50,000 IU/ml, designated as hi) and healthy donors (HD). (**A**) Gating strategies for CD4^+^ and CD8^+^ T cells and their subsets based on CD45RO and CCR7 expression; naïve (CD45RO^−^CCR7^+^), central memory (CM^;^ CD45RO^+^CCR7^+^), effector memory (EM^;^ CD45RO^+^CCR7^−^) and terminally differentiated effector memory (EMRA; CD45RO^−^CCR7^−^). Gating strategies for B cells and their subsets based on CD21 and CD27 expression; naïve (CD27^−^CD21^+^), resting memory (RM^;^ CD27^+^CD21^+^), active memory (AM; CD27^+^CD21^−^) and atypical memory (ATM; CD27^−^CD21^−^). (**B**) Frequencies of total CD4^+^, CD8^+^ T cells and total B cells among groups. (**C**) Proportions of the naïve, CM, EM and EMRA subsets of CD4^+^ and CD8^+^ T cells. (**D**) Proportions of the naïve, RM, AM and ATM subsets of B cells. Each data point represents an individual sample and horizontal line represents the median value. One-way-ANOVA/Kruskal-Wallis with Bonferroni/Dunn’s post-hoc tests for multiple comparison were performed for parametric or non-parametric data respectively. *p < 0.05, **p < 0.005.

To determine if CHB patients with varying level of serum HBsAg have differential phenotypic attributes of immune cell subsets, frequencies of T cells, B cells (Fig. 1A), monocytes, NK cells and Tregs were compared between the patients with HBsAg < 500 IU/ml (HBs^lo^) and HBsAg > 50,000 IU/ml (HBs^hi^). Healthy donors (HD) were included as reference. No differences in the proportion of total T cells, CD4^+^ and CD8^+^ T cells, B cells (Fig. 1B), monocytes, NK cells and Tregs (Supplemental Fig. S1) were found among the HBs^hi^, HBs^lo^ and HD groups. Further analysis of memory T cell subsets based on CCR7 and CD45RO expression showed no significant difference in the proportions of the naïve, central memory (CM), effector memory (EM) or terminally differentiated effector T cells (EMRA) between the HBs^hi^ and HBs^lo^ groups (Fig. 1C). However, CM CD4^+^ T cells increased significantly in both groups compared to HD, along with a proportional decrease in the naive subset (Fig. 1C). In contrast, the proportions of the naïve and CM CD8^+^ T cell subsets were similar among the groups (Fig. 1C). Analysis of memory B cell subset based on CD21 and CD27 expression also showed no difference in the proportion of the naïve, resting memory (RM), active memory (AM) and atypical memory cell (ATM) subsets between HBs^hi^ and HBs^lo^ groups (Fig. 1D). Only naïve B cell subset of HBs^hi^ were lower than those of HD. These data indicate that serum HBsAg levels had minimal impact on the global immune cell composition.

### PD-1 expression on CD4^+^ T cells is lower in HBs^lo^ patients

**Figure 2:**
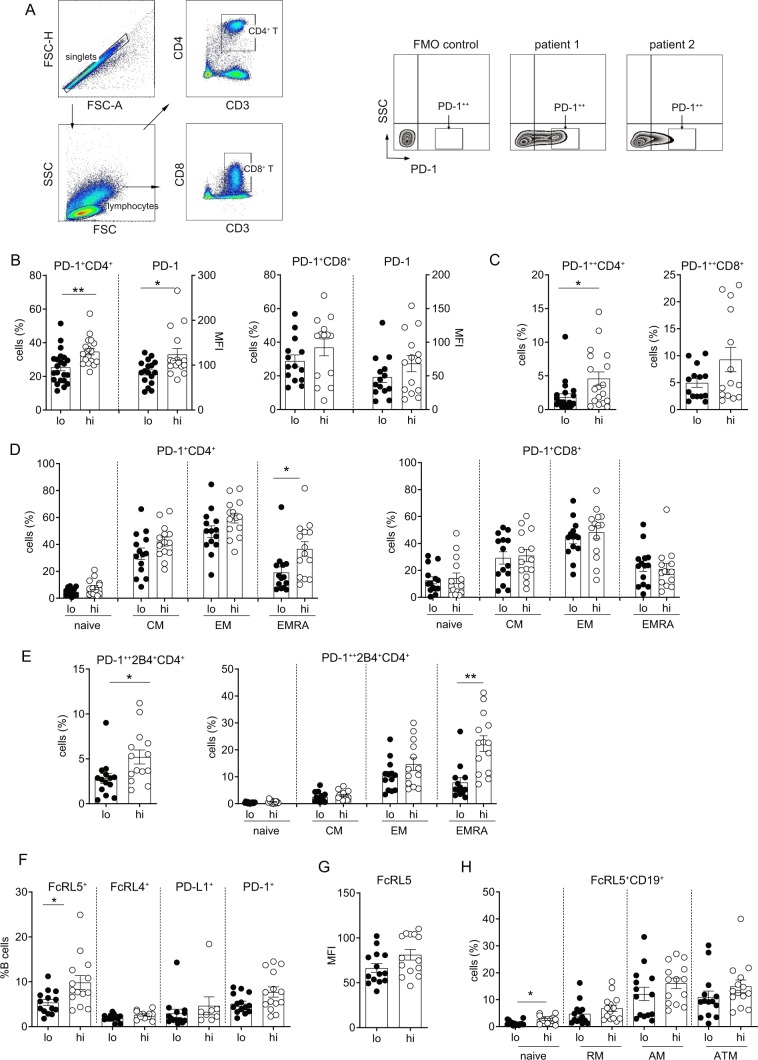
T cell and B cell phenotypic analysis by flow cytometry between the HBs^lo^ (designated as lo) and HBs^hi^ (designated as hi) groups. (**A**) Gating strategies for PD-1^+^ and PD-1^++ (high)^ on T cells. (**B**) Frequencies and MFI of total PD-1^+^ CD4^+^ or CD8^+^ T cells. (**C**) Frequencies of CD4^+^ and CD8^+^ T cells expressing high PD-1 (PD-1^++^). (**D**) Proportions of CD4^+^ or CD8^+^ T cell subsets expressing PD-1. (**E**) Frequencies of total CD4^+^ T cells and their subsets expressing both PD-1 an 2B4. (**F**) Frequencies of total B cells expressing FcRL5, FcRL4, PD-L1 or PD-1 and MFI of FcRL5^+^ B cells. (**G**) Proportions of B cell subsets expressing FcRL5. Each data point represents 1 sample and horizontal line represents the median value. Unpaired t test or Mann-Whitney U test were performed for parametric or non-parametric data respectively. *p < 0.05, **p < 0.005.

Chronic viral infection is associated with T cell exhaustion manifested by sustained expression of inhibitory receptors such as PD-1, 2B4 (CD244), CTLA4, LAG3, TIM3, CD160^22^. To determine if the exhaustion phenotypes are differentially manifested depending on serum HBsAg levels, PD-1 expression on T cells was compared between the HBs^hi^ and HBs^lo^ groups (Fig. 2A). Both %PD-1^+^ cells and PD-1 mean fluorescence intensity (MFI) on CD4^+^ T cells were significantly higher in the HBs^hi^ than HBs^lo^ group, but not on CD8^+^ T cells (Fig. 2B). Moreover, %CD4^+^ T cells expressing high levels of PD-1 (PD-1^++^), a phenotype associated with deeper exhaustion^23^, was also significantly higher in the HBs^hi^ than HBs^lo^ group, whereas no such difference was found for CD8^+^ T cells (Fig. 2C). Further analysis of PD-1 expression on T cell subsets revealed that PD-1 is expressed generally higher in the CM and EM subsets compared to the naïve and EMRA subsets of both CD4^+^ and CD8^+^ T cells regardless of HBsAg levels (Fig. 2D). However, significantly higher expression of PD-1 was observed on the CD4^+^ T cell EMRA subset in the HBs^hi^ compared to HBs^lo^ group (Fig. 2D). No such difference was found for the CD8^+^ T cell EMRA cells (Fig. 2D). Since co-expression of multiple inhibitory receptors sustains functional exhaustion of T cells^22,24^, 2B4 expression on T cells was also analyzed. While 2B4 expression on CD4^+^ T cells was not different between the groups (Supplemental Figs. S2-1), co-expression of PD-1 and 2B4 on CD4^+^ T cells was significantly higher in the HBs^hi^ compared to the HBs^lo^ group, with the most significant difference found in the CD4^+^ T cell EMRA subsets (Fig. 2E). Expression of these markers on T cells from healthy controls were lower when compared with HBs^hi^ group, while the differences between healthy and HBs^lo^ groups were not statistically different (Figs. S2–2). Collectively, higher serum HBsAg levels were directly associated with changes in the exhaustive phenotype of T cells, primarily in the CD4^+^ T cell compartment.

B cells associated with dysfunction and exhaustion also express an array of inhibitory molecules, including FcRL4, FcRL5, PD-1 and PD-L1. While both HBs^hi^ and HBs^lo^ groups expressed varying degree of these receptors (gating strategy, Supplementary Figs. 2 and 3, only FcRL5 expression was significantly different between the groups, while the rest of markers did not differ (Fig. 2F). CD19^+^ B cells expressing FcRL5 were present in significantly higher frequencies in the HBs^hi^ than HBs^lo^ group; however, no corresponding increase in FcRL5 MFI was detected (Fig. 2G). When analyzed further for %FcRL5^+^ B cell subsets, its expression on the naïve B cells was significantly higher in the HBs^hi^ than HBs^lo^ group, while other subsets showed same trend (Fig. 2H).

### HBV-specific polyfunctional CD4+ T cell responses were higher in HBs^lo^ patients

**Figure 3:**
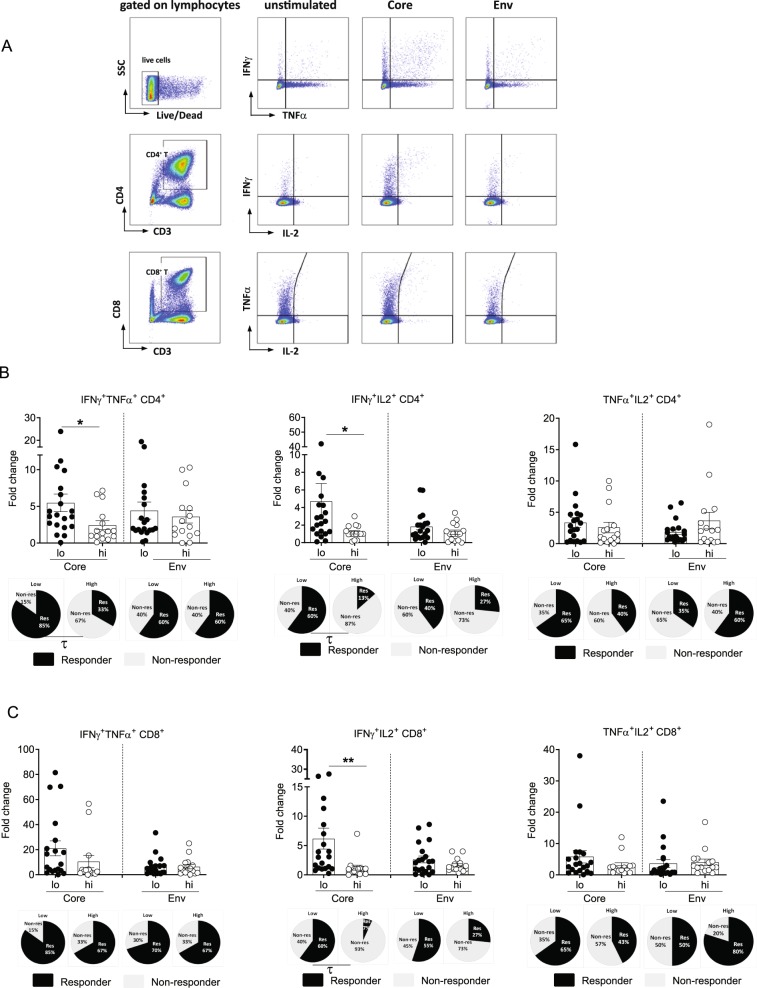
Flow cytometric analysis of HBV-specific CD4^+^ and CD8^+^ T cell responses between the HBs^lo^ (designated as lo) and HBs^hi^ (designated as hi) groups. (**A**) Gating strategies for intracellular cytokines IFNγ, TNFα and IL2 expression in CD4^+^ and CD8^+^ T cells. (**B**) Fold change in the frequency of CD4^+^ T cells or (**C**) CD8^+^ T cells secreting dual cytokines (IFNγ^+^TNFα^+^, IFNγ^+^IL2^+^ or TNFα^+^IL2^+^) following stimulation with HBcAg (Core) or HBsAg (Env) pooled peptides. Data were presented as fold change with respect to unstimulated control for individual samples. Each data point represents 1 sample and horizontal line represents the median value. Pi-charts represent the proportion of patients ≥2-fold change (Responder, Dark shade) and those <-fold change (Non-responder, light shade) in the measured parameters. Unpaired t test or Mann-Whitney U test were performed for parametric or non-parametric data respectively. *p < 0.05, **p < 0.005. χ^2^-test were performed to assess an association between serum HBsAg level and fold changes to HBV peptide stimulation. τ; p < 0.05 in χ^2^-test.

To determine the association between serum HBsAg levels and HBV-specific T cell responses, PBMCs from CHB patients of the HBs^hi^ (n = 15) and HBs^lo^ (N = 20) groups were stimulated with overlapping peptides of core (HBcAg) or env (HBsAg) antigens for 10 days. HBV-specific T cell responses were then assessed by fold change of %IFNγ, TNFα or IL-2 secretion of HBV peptide-stimulated CD4^+^ and CD8^+^ T cells (Fig. 3A). For 10-day cultures as performed in our study, proliferation of HBV-specific T cells takes place in an exponential manner, thus fold change in peptide stimulated condition with respect to unstimulated condition was deemed as appropriate representation of antigen specific response. Additional comprehensive analysis of %cytokine^+^ cells (single, double or triple^+^) in unstimulated and stimulated conditions is provided in the Supplementary Fig. 4C. In general, IFNγ and TNFα responses were dominant compared to IL-2. In addition, consistent with HBV core being more immunodominant, core-specific T cell responses were more readily detectable than env-specific responses (Fig. 3B,C). For CD4^+^ T cell responses, no significant differences in %core or env-specific CD4^+^ T cells secreting single cytokine (IFNγ, TNFα or IL-2) were observed between the two groups (Supplemental Fig. S4A). Since T cells producing multiple cytokines are associated with protective immune responses to numerous infectious diseases^25^, CD4^+^ T cells secreting 2 or more cytokines, defined as polyfunctional CD4^+^ T cell responses, were analyzed (Fig. 3A). CD4^+^ T cells secreting either IFNγ/TNFα or IFNγ/IL2 were significantly higher in the HBs^lo^ compared to HBs^hi^ group (Fig. 3B). The proportion of “responders”, defined as patients with ≥2-fold change in %cytokine^+^ cells, were also significantly higher in the HBs^lo^ than HBs^hi^ group for the core-specific polyfunctional CD4^+^ T cells (τ, p < 0.05; IFNγ^+^TNFα^+^, 85% vs. 33% respectively; IFNγ^+^IL2^+^, 60% vs. 13%, respectively) (Fig. 3B).

Although, there was no significant difference in the expression of inhibitory markers on CD8^+^ T cells between the groups (Fig. 2B–E), it is possible that polyfunctional CD8^+^ T cells may be differentiated depending on serum HBsAg levels. Similar with the observed CD4^+^ T cell responses, IFNγ and TNFα secretion was dominant compared to IL-2, in response to core rather than env stimulation. In addition, no significant difference in single cytokine (IFNγ or TNFα or IL2) production from %core-specific CD8^+^ T cells was found between the two groups (Supplemental Fig. S4D). However, polyfunctional response analysis showed that %core-specific IFNγ^+^IL2^+^ CD8^+^ T cells were higher in the HBs^lo^ compared to HBs^hi^ group, while there was no difference in %IFNγ^+^TNFα^+^ and %IFNγ^+^IL2^+^ CD8^+^ T cells between two groups (Fig. 3C). Furthermore, %responders in IFNγ^+^IL2^+^ CD8^+^ T cells was also significantly higher in the HBs^lo^ than HBs^hi^ group (τ, p < 0.05; IFNγ^+^IL2^+^, 60% vs. 7%, respectively) (Fig. 3C).

To test whether HBsAg levels impact global T cell response, we evaluated polyclonal T cell response by measuring cytokine production upon PMA/Ionomycin or anti-CD3/anti-CD28 stimulations. No significant differences in single or multi cytokine producing CD4+ or CD8+ T cells were observed between HBs^lo^ and HBs^hi^ groups (Supplementary Fig. S3). Therefore, HBV-specific T cell functions highlighted by polyfunctional T cell responses, are better preserved in patients with lower serum HBsAg levels, while such features are more readily presented in CD4^+^ T cells than in CD8^+^ T cells.

### Checkpoint blockade improves HBV-specific CD4^+^ T cell responses in HBs^lo^ patients

**Figure 4:**
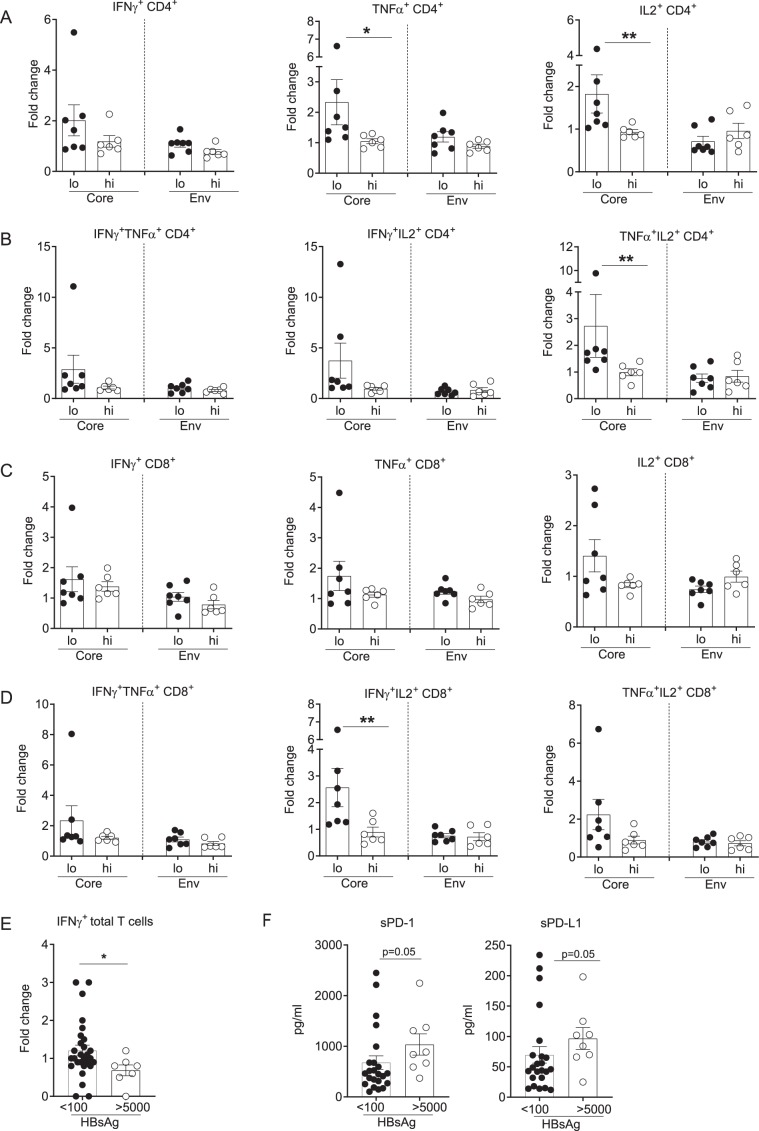
Analysis of HBV-specific T cell responses following checkpoint blockade with αPD-L1 antibody between the HBs^lo^ (designated as lo) and HBs^hi^ (designated as hi) groups. Fold change in the frequency of (**A**) single cytokine (IFNγ^+^, TNFα^+^ or IL2^+^) or (**B**) dual cytokine (IFNγ^+^TNFα^+^, IFNγ^+^IL2^+^ or TNFα^+^IL2^+^)-producing CD4^+^ T cells following stimulation with HBcAg (Core) or HBsAg (Env) pooled peptides with/without αPD-L1 by flow cytometric analysis. Fold change in the frequency of (**C**) single cytokine (IFNγ^+^, TNFα^+^, or IL2^+^) or (**D**) dual cytokine (IFNγ^+^TNFα^+^, IFNγ^+^IL2^+^ or TNFα^+^IL2^+^)-producing CD8^+^ T cells following stimulation with HBcAg (Core) or HBsAg (Env) pooled peptides with/without αPD-L1 by flow cytometric analysis. Data were presented as fold change by HBV peptide + αPD-L1 with respect to HBV peptide alone. (**E**) ELISpot analysis of total T cells secreting IFN*γ* following stimulation with HBcAg (Core) or HBsAg (Env) pooled peptides with/without αPD-L1. Data were presented as fold change by stimulation with HBV peptide +αPD-L1 with respect to HBV peptide alone in patients with HBsAg < 100 IU/ml and HBsAg > 5,000 IU/ml. (**F**) Comparison between patients with HBsAg < 100 IU/ml and HBsAg > 5,000 IU/ml for plasma soluble PD-1 and PD-L1 (sPD-1, sPD-L1) analyzed by Luminex. Each data point represent 1 sample and horizontal line represents the median value. Unpaired t test or Mann-Whitney U test were performed for parametric or non-parametric data respectively. *p < 0.05, **p < 0.005, ns; not significant.

Our data indicate that serum HBsAg levels are associated with PD-1 expression and HBV-specific T cell function. To determine if serum HBsAg levels also correlate with reversal of T cell exhaustion by checkpoint blockade using αPD-L1 antibody^6,26^, PBMCs from HBs^hi^ (n = 6) or HBs^lo^ (n = 7) groups were stimulated with overlapping peptides (HBcAg/HBsAg) with or without αPD-L1 for 10 days and HBV-specific T cell responses were analyzed by flow cytometry. The effect of αPD-L1 was presented as fold change of %cytokine^+^ T cells of HBV peptide-stimulated PBMCs in the presence of αPD-L1, with respect to peptide stimulation alone (Fig. 4A–D). Fold change in %core-specific CD4^+^ T cells secreting TNFα or IL-2 was significantly higher in the HBs^lo^ than in HBs^hi^ group, while IFN*γ* response was marginally different (Fig. 4A). In addition, αPD-L1 induced higher fold changes in HBcAg-specific, polyfunctional CD4^+^ T cell responses (TNFα^+^IL2^+^ CD4^+^ T cells; Fig. 4B). No such difference was found in env-stimulated CD4^+^ T cell responses (Fig. 4A,B). αPD-L1 did not induce fold change in CD8^+^ T cells secreting single cytokines regardless of viral antigen (Fig. 4C). However, as with the CD4^+^ T cell responses, polyfunctional CD8^+^ T cell responses were enhanced by αPD-L1, such that fold change in %HBcAg-specific, IFN*γ*^+^IL-2^+^ CD8^+^ T cells was significantly higher in the HBs^lo^ compared to HBs^hi^ group (Fig. 4D). Although the flow cytometric analysis was useful for delineating polyclonal T cell responses to checkpoint blockade, many patients still showed marginal responses to checkpoint inhibition, indicating the proportion of non-responders may obscure the significance of the overall responses. It was also possible that even lower HBsAg levels may be required for better responses to checkpoint blockade. To accommodate these possibilities, PBMCs from another patient cohort (Supplemental Table 2) divided into the HBs >5,000 IU/ml and HBs <100 IU/ml groups based on sample availability, were analyzed for IFN*γ*-secreting total T cell responses by ELISpot analysis (Fig. 4E and Fig. S5). Because these bio-samples were obtained through bioreclamation, less demographics and clinical data are known for this cohort, nonetheless we were able to identify subjects with very low (<100 IU/ml) HBsAg to supplement this study. Consistent with low HBsAg levels associated with better responses to checkpoint blockade, the patients with HBs <100 IU/ml showed significantly higher IFN*γ* responses than HBs >5,000 IU/ml (p = 0.02) (Fig. 4E). Interestingly, patients with HBs <100 IU/ml also had lower levels of soluble PD-1 and PD-L1 (sPD-1, sPD-L1) in their circulation compared to the HBs >5,000 IU/ml group (p = 0.05, Fig. 4F). These data indicate that driving HBsAg to even lower levels may result in better responses to checkpoint blockade.

### Checkpoint blockade improves HBsAg-specific B-cell responses

**Figure 5:**
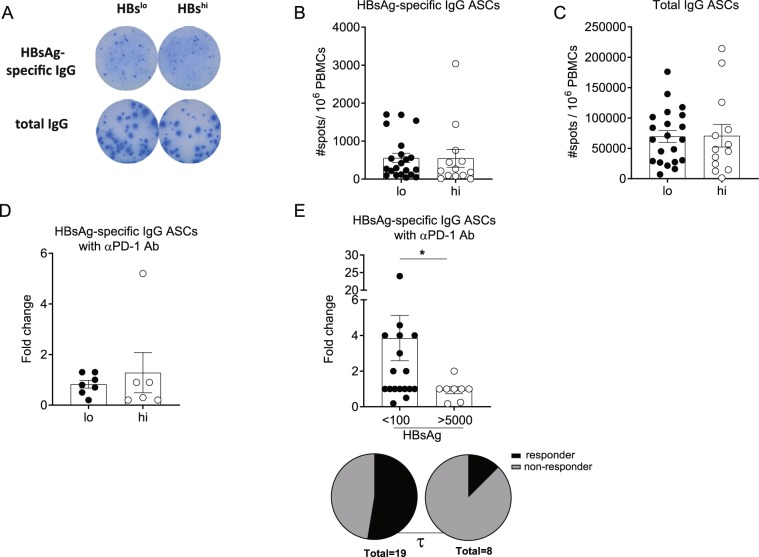
Analysis of HBsAg-specific B cell responses by HBsAg-specific ELISpot assay. PBMCs were stimulated with R848 and rIL-2 for 5 days and HBsAg-specific IgG as well as total IgG antibody-secreting cells (ASCs) were assessed by HBsAg-specific ELISpot assay. (**A**) Representative ELISpot image for HBsAg-specific or total IgG ASCs from the HBs^lo^ (designated as lo) or HBs^hi^ (designated as hi) patients. Number of (**B**) HBsAg-specific IgG and (**C**) total IgG ASCs per 10^6^ PBMCs from the HBs^lo^ (n = 21) and HBs^hi^ (n = 13) groups. (**D**) Fold change of HBsAg-specific IgG ASCs by R848 + αPD-1 antibody with respect to R848 stimulation alone, between the HBs^lo^ and HBs^hi^ groups. (**E**) Fold change of HBsAg-specific IgG ASCs by CpG-B + αPD-1 with respect to CpG-B stimulation alone, between patients with HBsAg < 100 IU/ml and HBsAg > 5,000 IU/ml by ELISpot assay. Pi-charts represent the proportion of patients ≥ 2-fold change (Responder, Dark) and those <-2-fold change (non-responder) between 2 groups. Each data point represents 1 sample and horizontal line represents the median value. Mann-Whitney U test were performed for statistical analysis. *p <0.05. χ^2^-test were performed to assess an association between serum HBsAg level and fold changes to αPD-1 antibody. τ; p < 0.05 in χ^2^-test.

It is possible that similar with HBV-specific T cell responses, B cell function in CHB patients may also be dysregulated in a HBsAg level-dependent manner. However, the number of HBsAg-specific as well as total IgG antibody-secreting cells (ASCs) detected by ELISpot assay following *in vitro* polyclonal stimulation were not different between the HBs^lo^ and HBs^hi^ groups (Fig. 5A–C). In addition, the fold changes in HBsAg-specific (Fig. 5D) as well as total IgG ASC responses (data not shown) by PD1/PD-L1 blockade using αPD-1^9^ were not different between the two groups. Similar with total IFN*γ* T cell responses to checkpoint blockade, it was possible that the sensitivity of B cell response to checkpoint blockade may require much lower serum HBsAg levels than the defined low value (500 IU/ml) of the current study. To this end, HBsAg-specific ASC responses to αPD-1 were compared between patients with HBsAg < 100 IU/ml and with >5,000 IU/ml (Fig. 5E). Interestingly, patients with HBsAg < 100 IU/ml group showed significantly higher fold change in HBsAg-specific IgG ASCs than those with HBsAg > 5,000 IU/ml (p = 0.03, Fig. 5E). The patients with HBsAg < 100 IU/ml also showed higher number of responders (≥ 2-fold) than those with HBsAg > 5,000 IU/ml (τ, p < 0.05). Therefore, while HBsAg-specific ASC responses were detectable in all CHB patients regardless of serum HBsAg (HBs^hi^ vs. HBs^lo^) levels, serum HBsAg levels <100 IU/mL may represent a cutoff that differentiates B cells’ plasticity to checkpoint modulation among CHB patients.

## Discussion

Functional cure of CHB infection, defined as off-treatment sustained HBsAg loss, is thought to be coupled with re-establishment of host immune control to HBV. T cell-mediated clearance of infected hepatocytes and antibody-mediated neutralization of virus is a hallmark of these anti-HBV immune responses. Thus, reduced HBsAg levels could manifest a gradient restoration of HBV-specific immune function in addition to alterations in overall immunophenotypes. In this study, we found that immune cell composition is largely unaffected by peripheral HBsAg levels and that HBV-specific immune responses were minimal in most CHB patients. However, HBV-specific immune responses of CHB patients with higher HBsAg levels (>50,000 IU/ml) is further impaired compared to patients with lower HBsAg levels (<500 IU/ml). These differences are likely in part a result of a more exhaustive (PD-1^++^ and PD-1^+^ 2B4^+^) phenotype in patients with HBsAg > 50,000 IU/ml. In addition, cytokine secretion (single and polyfunctional) to HBV peptide stimulation with checkpoint blockade with αPD-L1 antibody was significantly compromised in patients with higher HBsAg levels. Conversely, HBV-specific T and B cell responses to checkpoint blockade were even greater when HBsAg levels were very low (<100 IU/ml). Collectively, these data support the theory that reduction of HBsAg levels in CHB patients is accompanied by a concomitant increase in HBV-specific immunity. Although none of the patients in this study have reached functional cure, the current data suggests that patients with lower HBsAg could be “closer” to such an endpoint or possibly represent a population with a higher success rate in curative clinical trials aimed at restoration of natural immunity. With the lack of acceptable cure rates with current standard of care, definitive proof on the role of serum HBsAg levels in the potential for functional cure needs to wait on future studies utilizing combination curative therapies.

This study highlights a surprising and important correlation of HBsAg and HBV-specific CD4^+^ T cell responses. In fact, little correlation existed between HBsAg levels and peripheral CD8^+^ T cell responses and exhaustion phenotype. While it is known that cytotoxic function of CD8^+^ T cells is critical for resolution of HBV infection, it is possible that impairment of helper activities of CD4^+^ T cells is a central component of overall HBV-specific immune dysregulation in CHB. Reduced HBV-specific CD4^+^ T cell responses would result in impaired HBV-specific B cell responses and seroconversion as well as defective CD8 T cell responses. At first glance, it seems contradictory that little association was found between CD8^+^ T cells and HBsAg levels especially in light of recent reports that PD-1^+^CD8^+^ T cells correlate with lack of liver flare upon NA cessation^27^. However, the majority of CHB patients in this study have HBsAg ~1000 IU/ml regardless of NA treatment/cessation and the presence of PD-1^+^CD8^+^ T cells is associated with the prevention of liver flare rather than long-term viral control and functional cure. Therefore, it is possible that PD-1 expression on CD8^+^ T cells could decrease during sustained responses after NA cessation, as HBsAg levels gradually decline. Collectively, these results together with our current findings may indicate the longitudinal immunological events *en route* to immune control, such that as HBsAg levels decrease, HBV-specific immunity begins the “re-awakening” process by first restoring specific, polyfunctional CD4^+^ T cells, followed by restoration of functional CD8^+^ T cells. Finally, given the HBsAg-specific B-cell responses to PD-1/PD-L1 blockade are detectable in patients with very low HBsAg levels, restoration of B cell humoral responses could represent the final cascade of immunological events leading to full immune memory responses. Consistent with this idea, animal models have shown that HBV-specific CD4^+^ T cells function as a putative master regulator of CD8^+^ T cell antiviral responses^28^.

Whether higher antigen specific functional response in patients with low HBsAg levels reflects presence of higher levels of HBV specific T cells in this group cannot be known from our data, since we did not measure tetramer biding cell *ex vivo*. The cytokine response likely reflects the initial frequency of antigen specific cells and/or functional response of those cells to antigen. Since we have used short-term cultures with HBV peptides to measure antigen specific response, better response in this group suggests that HBsAg levels in the least impact proliferative response of antigen specific T cells. Why CD4^+^ T cell responses would be more affected by high serum HBsAg levels than CD8^+^ T cells isn’t clear, but some clues can be obtained from other chronic viral infection models, with high systemic antigenemia of chronic lymphocytic choriomeningitis virus (LCMV) infection. High levels of soluble viral antigens can readily form immune complexes (ICs) and physically block FcgR on antigen-presenting cells (macrophages and DCs) and impair antigen presentation to CD4^+^ T cells^29,30^. Such presence of circulating ICs at the expense of virus-specific high affinity Ab responses have been documented in other viral infections including human immunodeficiency virus (HIV), hepatitis C virus (HCV)^31–33^ and HBV^34^. Why αPD-1 blocking was mostly beneficial in patients with lower HBs levels even though they had lower levels of PD-1 expression than HBs^hi^ patients can be explained by the heterogenous nature of PD-1 expressing cells. Expression of multiple inhibitory receptors on PD-1+D-CD4 T cells from HBs^hi^ patients (2B4 was shown in our study) possibly requires additional checkpoint blocks for detectable improvement in cytokine response. Additionally, lower PD-1 expression level defines cell populations that can better be rescued by checkpoint blocking. PD-1^hi^ or PD-1^++^ subsets, identified as terminally exhausted and not amenable to rescue by αPD-1^35^, was higher in our patients with high HBsAg levels.

There are few limitations of this study that should be outlined. The sample sizes are small for being able to make full conclusions applicable for all CHB patients, especially considering the large patient heterogeneity that exists in the general CHB population. Future studies engaging CHB patients whose HBsAg levels are in a continuous spectrum, including those who have achieved functional cure, are needed to further apply our findings to real-world clinical settings. Additionally, since HBsAg can be derived from cccDNA or integrated DNA, it remains possible that such distinction could interfere the current findings. It remains unknown if HBsAg levels are associated with HBV polymerase or X protein as there is increasing evidence on their contribution to the overall antiviral T cell responses^27,36^. Our current studies were not able to assess the breadth of HBV-specific T cell responses due to limited availability of the clinical specimen materials. Also, while the current study was focused on the role of HBsAg levels on HBV immune responses, there are other biomarkers that could also be correlative to HBV-specific immune function. It is known that HBcrAg levels are associated with HBeAg seroconversion, suggesting a possible correlation of this biomarker with HBV immunity^37^. Future studies will be focused on expanding the observations in this report to other potential peripheral and intrahepatic biomarkers. Finally, our investigation was limited to peripheral blood where HBV specific T cells are present in very low frequencies in CHB patients. Intrahepatic evaluation of HBV specific responses is likely to provide a clearer picture of HBsAg role in impairing antiviral CD8^+^ T cell response, since hepatic CD8^+^ T cell responses are known to correlate with HBV control^38^.

Currently, there are several therapeutic strategies in clinical development designed to directly reduce serum HBsAg levels. Our current findings suggest that subjects with pre-existing low HBsAg levels may be more poised to “re-awaken” HBV-specific immune responses either by antiviral or immunotherapeutic approaches or a combination approach. It is left to be determined if therapeutic strategies to directly drive serum HBsAg to low levels will shift CHB patient immune potential closer to cure, however several clinical trials are ongoing to test this hypothesis. With this said, the general consensus in the field is that lowering HBsAg levels will be a much needed step in the evolution of curative therapies^39^. With the heterogeneity of CHB patients as a main challenge for designing clinical trials, a better understanding of the underlying immune potential of CHB patients could provide a chance to stratify patients based on potential curative responses. As a starting point for such stratification, we provide in this study, evidence that serum HBsAg levels could be used as a surrogate biomarker for the competency of HBV-specific immunity in CHB patients. It is likely that functional differences exist in multiple immune compartments in CHB, spanning from innate to adaptive immunity. Therefore, further studies investigating multidimensional immune function and phenotypes associated with HBsAg levels could pave the way to better designing immunotherapies for CHB.

### Ethics statement

All participants signed informed consent approved by the University of Maryland School of medicine at the time of screening and enrollment and all samples were anonymized. All methods utilized for this study were performed in accordance with the relevant guidelines and regulations.

## Supplementary information

Supplementary Information.
