# Publisher Correction: Circulating serum HBsAg level is a biomarker for HBV-specific T and B cell responses in chronic hepatitis B patients

**DOI:** 10.1038/s41598-020-62445-6

**Published:** 2020-03-31

**Authors:** Jin Hyang Kim, Alip Ghosh, Natarajan Ayithan, Sara Romani, Arshi Khanam, Jang-June Park, Rene Rijnbrand, Lydia Tang, Michael J. Sofia, Shyam Kottilil, Chris B. Moore, Bhawna Poonia

**Affiliations:** 1 Arbutus Biopharma Corporation, 701 Veterans Circle, Warminster, Pennsylvania 18974 United States; 2 Institute of Human Virology, University of Maryland School of Medicine, Baltimore, MD 21201 United States

Correction to: *Scientific Reports* 10.1038/s41598-020-58870-2, published online 04 February 2020

Supplementary Tables 1 and 2 are omitted from the Supplementary Information which accompanies this Article. Tables S1 and S2 appear below.

**Table S1:** Demographic details of cohorts of HBs^lo^ and HBs^hi^ CHB patients and healthy subjects.

	CHB (HBs^lo^)	CHB (HBs^hi^)	healthy donors (HD)
Serum HBsAg levels (IU/ml)	<500	<50,000	N/A
number of subjects	23	16	10
median age (years) (range)	54 (31–80)	35 (26–60)	37 (63–27)
Gender (M/F)	20/3	12/4	6/4
Ethnicity (Asian/African/Caucasian)	15/5/3	5/11/0	2/0/8
HBV DNA (log IU/ml) (mean ± SD)	2.07 ± 1.04	4.43 ± 2.36	N/A
HBeAg (-ve/+ve/UA)	20/2/1	12/4/0	N/A
anti-HBeAb (-ve/+ve/UA)	5/14/4	5/6/5	N/A
median ALT (U/L) (range)	31.0 (9.0–542.0)	41.0 (14.0–536.0)	N/A
median AST (U/L) (range)	26.0 (15.0–430.0)	39.5 (17.0–259.0)	N/A

SD: standard deviation, UA: Unavailable, ALT: alanine transaminase, AST: aspartate transaminase, N/A: Not applicable.

**Table S2:** Details of subjects in second cohort of CHB patients.

	CHB HBsAg <100 IU/ml	CHB HBsAg >5,000 IU/ml
number of subjects	23	8
median age (years) (range)	52 (34–71)	52 (39–72)
Gender (M/F)	15/8	4/4
HBeAg (-ve/+ve/UA)	14/1/8	1/1/6

UA: Unavailable.

