# Supplementary material for: Circulating serum HBsAg level is a biomarker for HBV-specific T and B cell responses in chronic hepatitis B patients
Source: Sci Rep. 2020 Feb 4;10:1835. doi: 10.1038/s41598-020-58870-2 (PMC7000714; doi:10.1038/s41598-020-58870-2)

Supplementary Fig S1

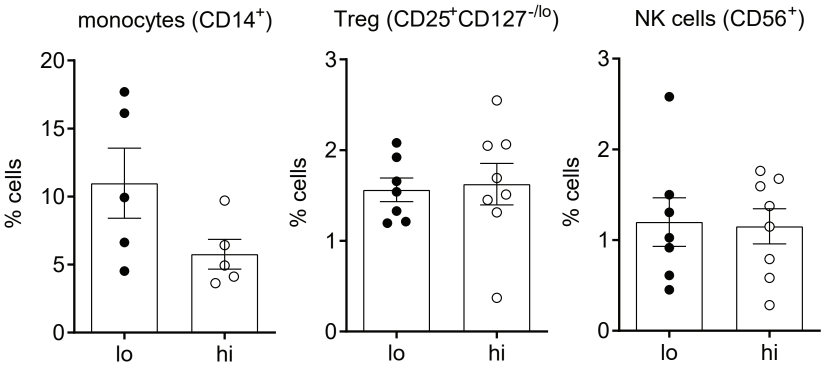

Supplementary Fig S2-1

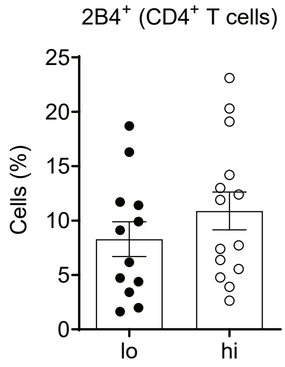

S2-2

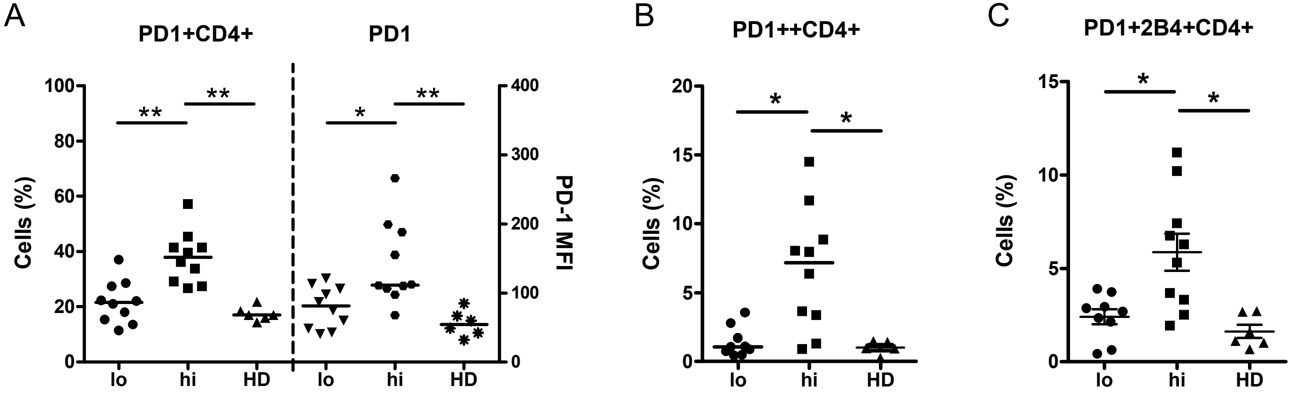
A
PD1+CD4+
PD1
Cells (%)
PD-1 MFI
100
80
60
40
20
0
400
300
200
100
0
**
**
*
**
lo
hi
HD
lo
hi
HD
B
PD1++CD4+
Cells (%)
20
15
10
5
0
*
*
lo
hi
HD
C
PD1+2B4+CD4+
Cells (%)
15
10
5
0
*
*
lo
hi
HD

Supplementary Fig S2-3

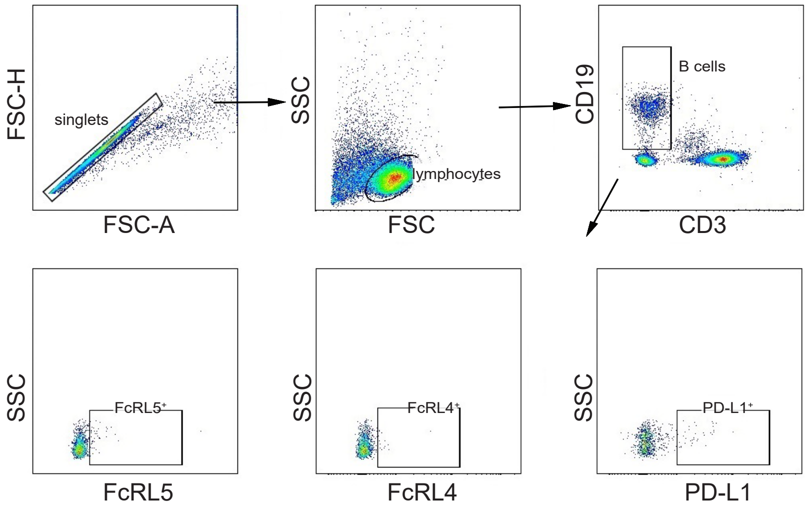

Supplementary Figure S3

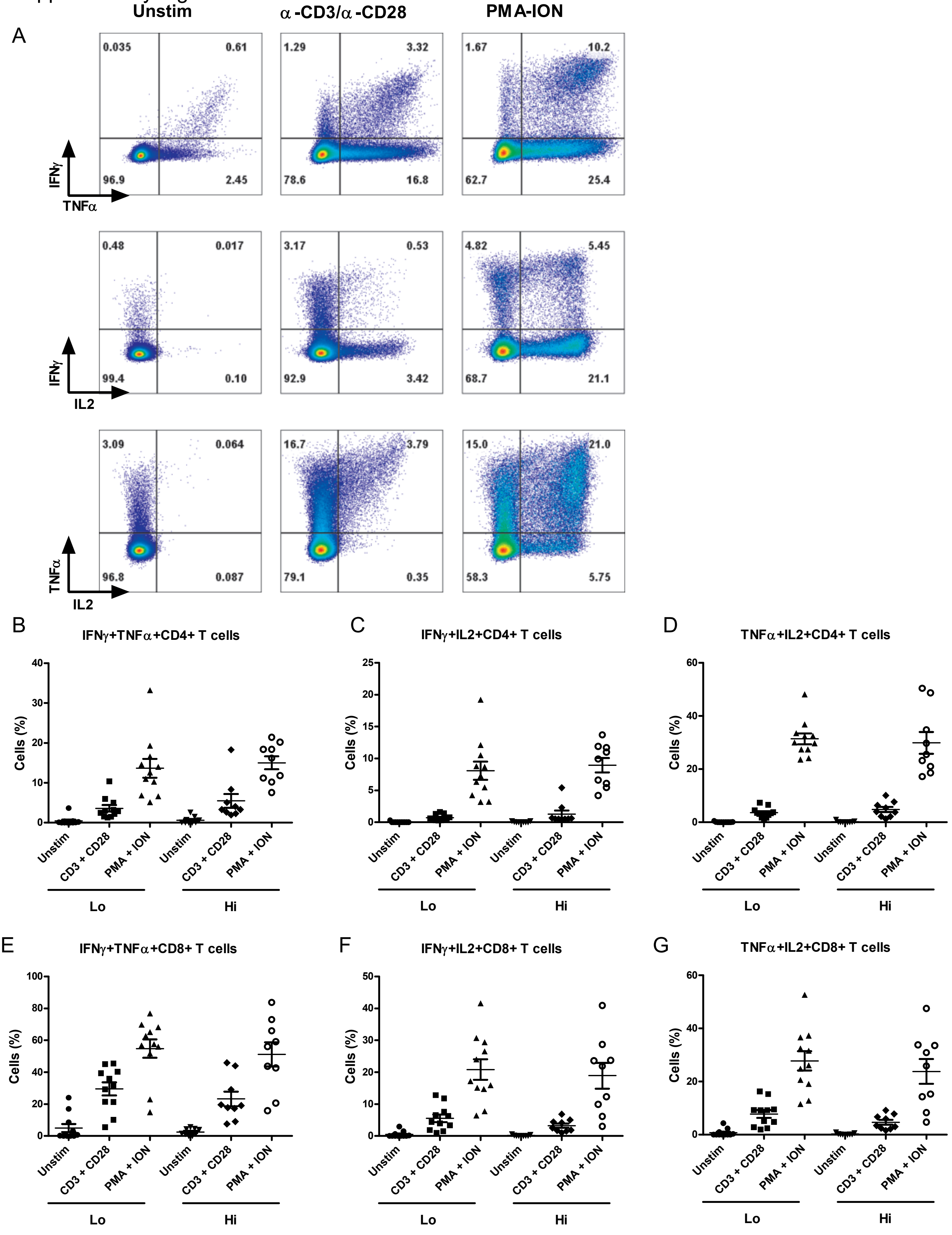

Supplementary Fig S4

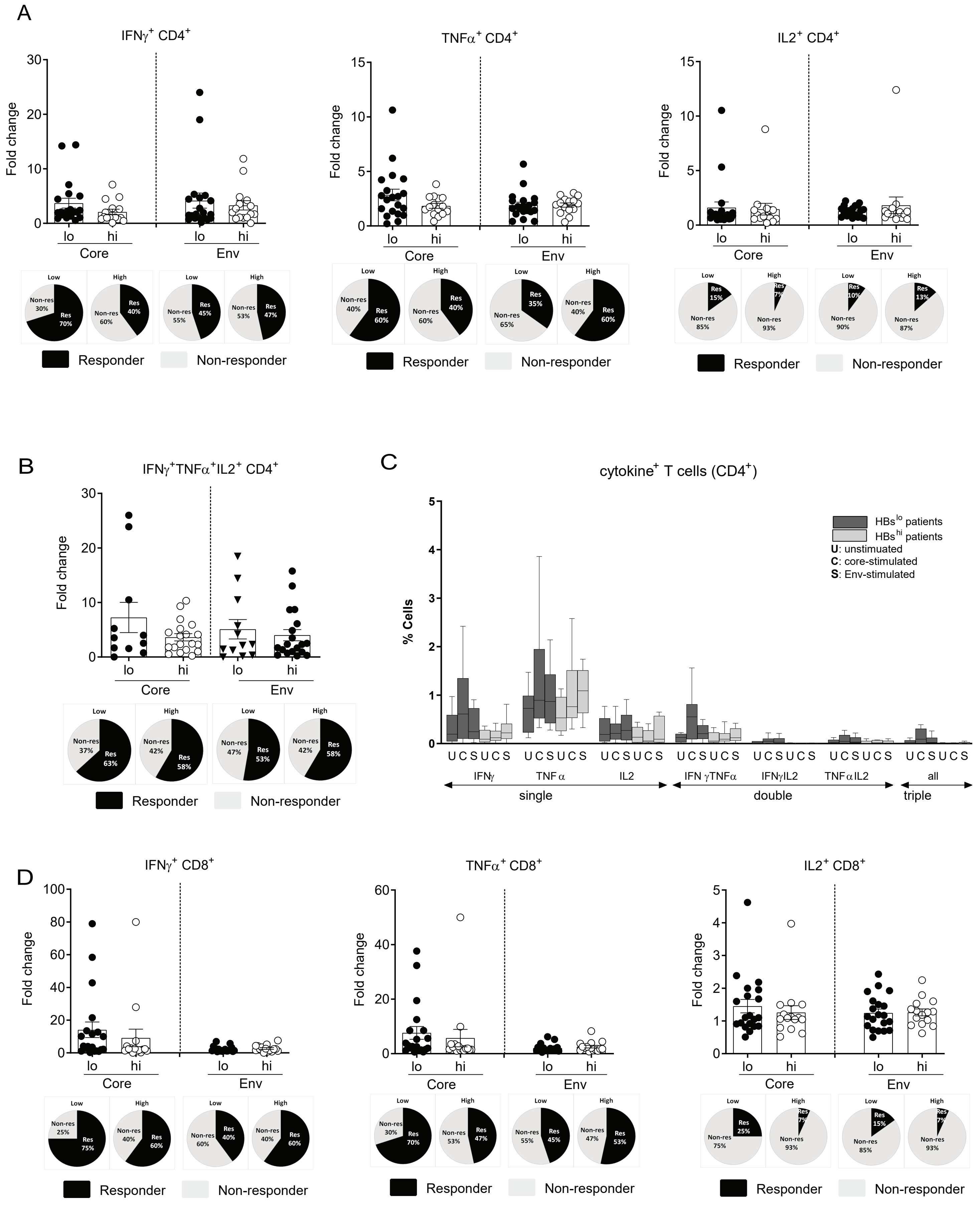

Supplementary Figure S5

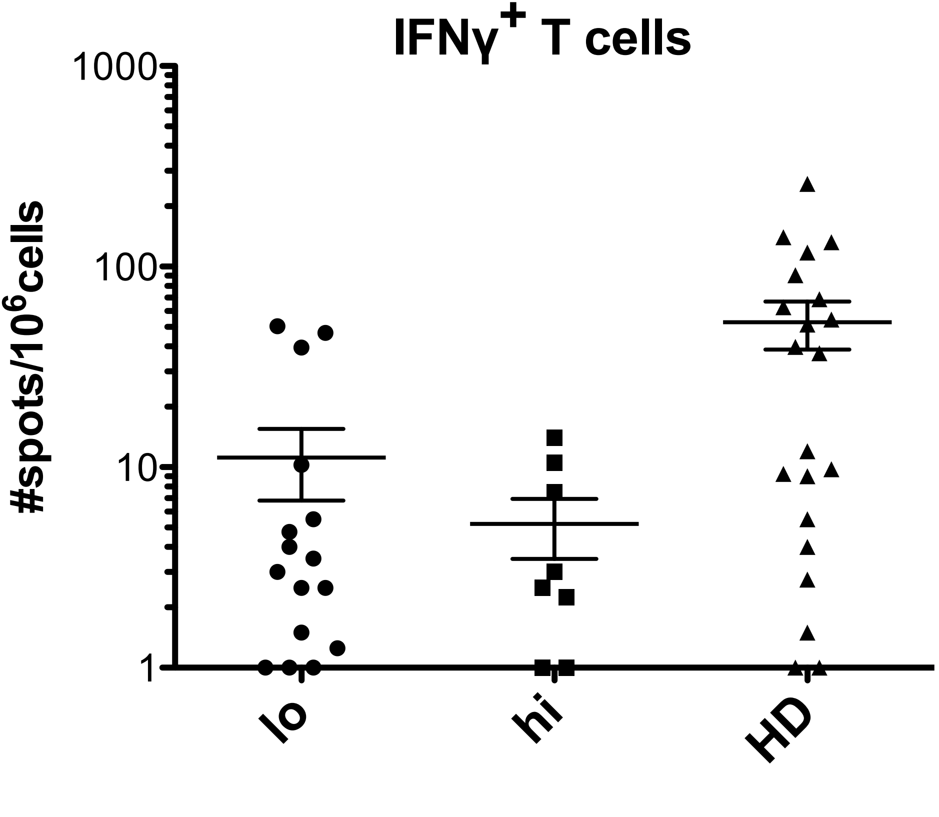

Supplement: Supplementary file 1 — Supplementary Information. [file 41598_2020_58870_MOESM1_ESM.pdf]
